# Prevalence and Management of Transfusional Iron Overload in Syrian Beta Thalassemia Major Patients Pre and during the Syrian Conflict

**DOI:** 10.1155/2023/8911518

**Published:** 2023-09-14

**Authors:** Hanan Touma, Lama A. Youssef, Lana Al-Salhi, Wouroud Ismail Al-khalil, Khawla AlKeba

**Affiliations:** ^1^Program of Clinical and Hospital Pharmacy, Department of Pharmaceutics & Pharmaceutical Technology, Faculty of Pharmacy, Damascus University, Damascus, Syria; ^2^University of Kalamoon, Deir Atiyah, Damascus, Syria; ^3^National Thalassemia Center, Homs, Syria; ^4^Al-Ahli Hospital, Homs, Syria

## Abstract

**Objectives:**

The primary aim of this study was to evaluate the prevalence of iron overload and the real-world clinical effectiveness of the iron chelation therapies (ICTs) in Syrian patients with transfusion-dependent beta thalassemia major (BTM) prior to and during the ongoing Syrian conflict.

**Methods:**

This single-center, two-stage observational study was conducted at Homs National Thalassemia Center (HNTC) prior to (2009) and during (2019) the armed conflict. The prevalence and the severity of iron overload, as well as the effectiveness of four iron chelation regimens, were assessed using serum ferritin (SF) concentrations as a means of monitoring in two cohorts of BTM patients receiving deferoxamine (DFO), deferiprone (DFP), deferasirox (DFX), or a combination of DFO and DFP therapy in both years. Statistical analyses encompassed one-way ANOVA, Kruskal-Wallis, Mann–Whitney *U*, and chi-square (*χ*2) tests for the comparisons of the variables and the frequencies between the two cohorts and subgroups.

**Results:**

We included all eligible BTM patients at HNTC in 2009 (*n* = 205) and 2019 (*n* = 172). Only 84 patients from the 2009 cohort were accessible in 2019. Our findings revealed that 98% and 89% of the patients had iron overload (*SF* ≥ 1500 ng/mL) and comparable elevated median SF concentrations (3868 and 3757 ng/mL) in 2009 and 2019, respectively (*P* = 0.275). Furthermore, patients on DFO demonstrated the poorest control of iron overload and the highest SF concentrations (4319 and 5586 ng/mL), whereas those on DFX achieved superior outcomes and the lowest SF concentrations (3355 and 2152 ng/mL) in both years. Twenty-six patients from the 2019 cohort received no ICT for six years (from 2012 to 2018) and experienced extremely severe iron overload with SF levels ranging between 4481 and 16,000 ng/mL.

**Conclusions:**

Our findings prove a high prevalence of iron overload and suboptimal chelation outcomes in Syrian BTM patients, both prior to and during the ongoing armed conflict, despite the provision of free ICTs at HNTC. Poor adherence and older age of patients may explain the unfavorable outcomes of DFO and (DFO+DFP) regimens, whereas younger age and higher socioeconomic status may have contributed to the lowest SF and superior outcomes in patients on DFX. This study also demonstrates the crucial role of the National Thalassemia Centers, namely HNTC, in providing health services to BTM patients in times of peace and conflict.

## 1. Background

The preconflict population of Syria was estimated to be approximately 22 ± 0.5 million individuals, featuring a diverse ethnic composition consisting of a majority of Arab Descent, as well as minorities of Kurds, Armenians, Turkmens, and others, alongside over 500 thousand Palestinian refugees [1].

Thalassemia, a prevalent autosomal abnormality in the Mediterranean and Near East Region, is the predominant genetic disorder in the Syrian population, particularly in the outskirts of Damascus, Golan Heights, Aleppo, and the Syrian Coast, with approximately 5% of the population carrying the beta thalassemia trait [2]. The high prevalence of thalassemia in Syria contributes to inadequate prevention strategies as well as cultural and religious norms, including a high rate of consanguineous-marriages, particularly in the rural areas of the country [3].

Homozygosity or compound heterozygosity of *β*-thalassemia defects leads to transfusion-dependent *β*-thalassemia major (BTM), which requires chronic transfusion therapy to prevent death before the age of five [4]. However, long-term transfusion therapy leads to secondary iron overload-associated complications, particularly myocardial and hepatic siderosis and endocrinological dysfunction [5].

The timely initiation of chelation therapy represents the optimal approach to prevent iron accumulation and iron overload complications, and its effectiveness is linked to lower morbidity and mortality rates [6]. Deferoxamine mesylate (DFO) is the most widely used chelation agent administered subcutaneously or intravenously for over 8 to 12 hours up to seven days per week in adults and children at least 3 years old [7]. Deferasirox (DFX) and deferiprone (DFP) are two oral chelation agents initially introduced as second-line treatment in children with BTM aged at least 6 years or patients in whom DFO is contraindicated or inadequate [8]. Combinations of parenteral DFO with oral DFP [7, 9] or DFX [10] have been successfully implemented [11]. The selection of chelation therapy is influenced by various factors, including local clinical guidelines, practitioner discretion, patient's age, individual iron profile, iron consumption, cardiac and hepatic iron deposition, and heart failure [12].

The utilization of serum ferritin (SF) as a means of monitoring iron overload in BTM has been established due to its cost-effectiveness, convenience, well-established nature, and correlation with body iron stores [13]. Persistently high levels of SF exceeding 2,500 ng/mL have been associated with an increased risk of cardiac disease and mortality [8, 14, 15], whereas lower SF levels ranging from 500 to 1500 ng/mL are indicative of relatively decreased risk [8, 16].

The National Thalassemia Program in Syria was established in 1997 with the aim of providing regular blood transfusion, ICTs, and other related health services to all registered thalassemia patients through a network of national centers. However, despite the Syrian Ministry of Health's pledge to the provision of free ICTs to all BTM patients, efficient monitoring of therapeutic outcomes has been lacking, adherence to chelation therapy has never undergone a thorough evaluation, and measures to improve compliance have not been taken. These drawbacks in the management of iron overload resulted in significant morbidity and mortality rates in BTM patients, with cardiac complications being the primary cause of mortality [17].

The Syrian conflict, now in its twelfth year since its eruption in 2011, has had a profound and devastating impact on the lives of Syrians, resulting in the internal displacement of more than 6.1 million individuals and the exodus of 5.6 million refugees. The Syrian healthcare system has been severely damaged, with up to 50% of healthcare facilities being damaged and up to 70% of healthcare workers having fled the country due to the ongoing violence and economic and social sequels of the war. This has placed a considerable strain on the remaining medical professionals, exacerbating the already challenging situation [18, 19]. The consequences of the Syrian conflict on the healthcare system had particularly detrimental effects on thalassemia patients, especially those who were displaced or besieged in the armed conflict territories. The disruption of blood transfusion accessibility and iron-chelation therapies due to the war and later sanctions, along with the challenges associated with monitoring of therapeutic outcomes, has significantly compromised the quality of care available to this vulnerable population.

The National Thalassemia Program registry has reported a total of 214 newborn infants with BTM in 2018. Furthermore, as of March 2019, a total of 4,677 patients have been registered to receive chronic lifelong blood transfusion and concurrent chelation therapy at the Program's satellite centers positioned in the twelve Syrian governorates under government control [20].

The Syrian Ministry of Health (MOH) has maintained a well-established system for the management of emergency or strategic blood supplies prior to and during the armed conflict. National Thalassemia Centers, as well as all public hospitals, were mandated to provide blood transfusions to all Syrian BTM patients [20]. However, in the wake of the violent struggle in the city of Homs, the primary obstacle that encumbering the MOH's provision of blood supplies was the safety concerns associated with the physicians, nurses, paramedical staff, and patients' transportation to and from these medical facilities.

This study is aimed at evaluating the prevalence of poor chelation outcomes and secondary hemosiderosis and assessing the effectiveness of the different iron chelation regimens provided by the National Thalassemia Program to Syrian BTM patients at HNTC during times of peace (2009) and during the Syrian conflict (2019).

## 2. Subjects and Methods

### 2.1. Study Design and Subjects

This study was designed as a single-center, two phase observational study. The research team conducted a comprehensive review of the medical records of all patients who attended HNTC to receive blood transfusions and ICTs in 2009 and 2019. The data collected included information on patients' diagnoses, history of blood transfusion, iron chelator regimen, and SF concentration values.

The inclusion criteria encompassed male and female patients attending HNTC with a confirmed diagnosis of BTM, aged three years or older, on any regimen of iron-chelation therapy, and with SF levels measured on at least two occasions within a 12-month period. The diagnosis of BTM was established based on the following criteria: microcytic hypochromic anemia, nucleated red blood cells on peripheral blood smear, and hemoglobin analysis that revealed absence of hemoglobin A (HbA), fetal hemoglobin (HbF) of 90-96%, and (HbA2) of 4-10% [8].

The exclusion criteria included the presence of hemoglobinopathies other than BTM, lack of any iron-chelation therapy, refusal to undergo treatment, or unavailability of SF concentration records.

The present study was granted approval by the Scientific Research Bioethics Committee at the Faculty of Pharmacy, Damascus University. Informed written consents were obtained from all adult patients and the guardians of children following the provision of comprehensive information regarding the study's aims and methodologies coupled, and assurance of the confidentiality of the collected personal data.

DFO monotherapy (subcutaneously infused at 25-50 mg/kg over 8-10 hours a day for 5-7 days per week) was the only iron chelator provided by all National Thalassemia Centers, including HNTC, until 2008, when oral chelators (DFP and DFX) were introduced. Since then, patients who demonstrated a poor response to DFO monotherapy were treated with DFP (at 75-100 mg/kg/day) or DFX (at 20-40 mg/kg/day) monotherapy or a combination therapy with DFO and DFP. The recommended regimen, dosage of each chelator, and frequency of DFO infusions were initially adjusted based on the SF levels and subsequently maintained based on clinical features, echocardiogram findings, side effects, and patient preferences. These modifications were made by a hematologist in accordance with the guidelines established by the UK Thalassemia Society for the clinical management of children and adults with thalassemia [8].

### 2.2. Endpoints and Statistical Analyses

The study used SF concentrations to evaluate the efficacy of the four-chelator regimens, with a target SF of 1500 ng/mL [8, 15, 16]. Factors that influence SF concentrations, such as chronic alcohol consumption, metabolic syndrome, obesity, diabetes, malignancy, acute or chronic infection, chronic inflammatory disorder, and viral hepatitis, were ruled out via interviews and clinical assessments performed by health care providers. C-reactive protein (CRP) was used as a biomarker to rule out infection and inflammatory conditions.

Iron overload was classified as mild, moderate, or severe based on the following arbitrary cutoff values: mild (1500-2500 ng/mL), moderate (>2500 ng/mL and ≤4000 ng/mL), or severe (>4000 ng/mL) [8]. The severity of iron overload was considered improved if patients achieved therapeutic outcomes, or if the degree of iron overload decreased from severe-moderate to moderate-mild.

Statistical analyses were performed using Prism GraphPad®, version 9, with the Kolmogorov-Smirnov test used for normality testing of continuous variables. Normally distributed numeric variables were expressed as *mean* ± *standard* deviation (SD), while variables that are not normally distributed were expressed in terms of median and range. One-way ANOVA, Kruskal-Wallis, and Mann–Whitney *U* tests were used to compare means and medians of different continuous variables between the two cohorts. The chi-square (*χ*2) test was used to compare the frequencies of qualitative variables among the different groups. A *P* value less than 0.05 was considered statistically significant.

## 3. Results

### 3.1. Patients' Characteristics

**Figure 1:**
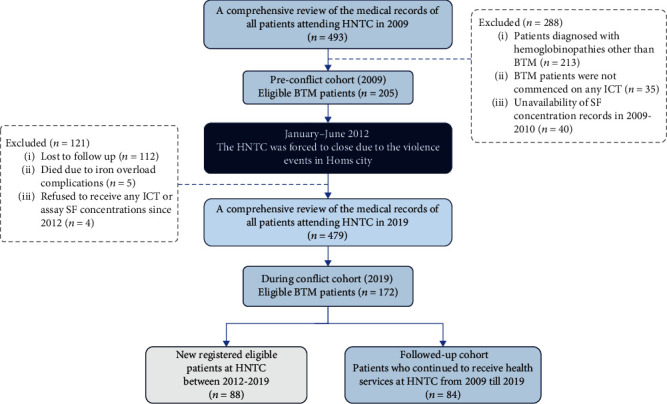
A flow diagram of the selection process of the 2009 (preconflict) and 2019 (during conflict) cohorts.

This observational study included two discrete cohorts (2009 vs. 2019). The medical records of all patients who attended HNTC in each year underwent rigorous examination to ensure the accuracy and reliability of the collected data. Out of the 493 and 479 total numbers of patients with hemoglobinopathies registered at the HNTC in 2009 (prior to the conflict) and 2019 (during the conflict), respectively, 205 and 172 patients were deemed eligible for inclusion in this study. Excluded patients were those with hemoglobinopathies other than BTM, those who were not commenced on iron chelation therapy, or those who had no recorded measurements of SF (as shown in Figure 1).

The median age of participants in the pre- and during conflict study populations was 14 and 10 years, respectively, and the male-to-female ratio was approximately 50 : 50 in both cohorts. Blood transfusions were given to patients in both cohorts at a frequency of two to three times per month, and the pre-transfusion median hemoglobin levels were 8.7 and 8.1 g/dL in the pre- and during-conflict cohorts, respectively.

Out of the 205 BTM patients at HNTC in 2009 (prior to the conflict), 39% received DFO monotherapy, 30% were given oral DFX, 21% were assigned a combination of DFO and DFP, while only 10% received oral DFP. However, in 2019, the percentage of patients on DFO monotherapy dropped to only 16.3%, whereas 29.1% of patients were given the combination regimen DFO+DFP, and a higher percentage (29.1%) of patients received DFP monotherapy. It is worth mentioning that the economic sanctions imposed on the country during the Syrian conflict disrupted the supply of medications and negatively affected the availability of oral DFX at the National Thalassemia Centers in 2019, with only (12.2%) of patients able to afford it. Moreover, a significant percentage (15.1%) of the patients had not received any ICT for six years, spanning from 2012 to 2018, due to their being trapped in the armed conflict areas. These patients have been identified as a distinct group, labeled as no ICTs for six years (2012-2018). The characteristics of patients in each cohort are summarized in Table 1.

Out of the 205 patients who attended HNTC in 2009 (the preconflict cohort), only 84 individuals resumed attendance HNTC in 2019. The remaining 121 patients were lost to follow-up or had succumbed due to violence events or complications of iron overload.

### 3.2. Comparison of SF Concentrations and Prevalence of Iron Overload between the 2009 (Preconflict) and 2019 (during Conflict) Cohorts

**Figure 2:**
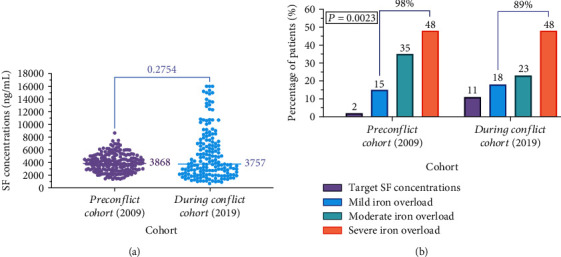
Comparison of SF concentrations (a) and prevalence and severity of iron overload (b) between the 2009 (preconflict) and 2019 (during conflict) cohorts.

The median SF concentrations were remarkably high and comparable (3868 ng/mL and 3757 ng/mL) in the 2009 and 2019 cohorts, respectively (*P* = 0.275), (Figure 2(a). Evidently, the vast majority of patients in both cohorts had iron overload (98% and 89% in 2009 and 2019, respectively). Patients with severe iron overload constituted almost half of each cohort (48%). Nevertheless, a significantly higher percentage (11%) of patients in the 2019 (during conflict) cohort achieved target SF concentrations or had mild iron overload (18%), compared with 2% and 15%, respectively, in the 2009 (preconflict) cohort (*P* = 0.0023, Figure 2(b)).

### 3.3. The Effectiveness of the Four Iron Chelation Regimens in the 2009 (Preconflict) versus 2019 (during Conflict) Cohorts

**Figure 3:**
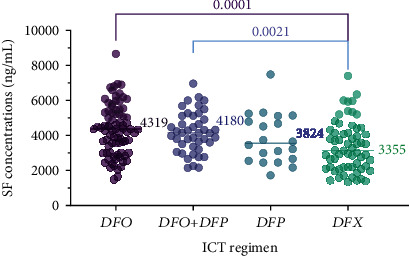
Comparison of SF concentrations (ng/mL) according to the administered ICT regimen in the preconflict (2009) cohort; patients on DFO had the highest median SF concentrations (4319 ng/mL), whereas those on DFX had the lowest (3355 ng/mL), while no statistically significant differences were observed in the median SF concentrations between patients on other ICT regimens.

In the 2009 (preconflict) cohort, patients on DFX monotherapy had significantly lower average SF concentrations (3355 ng/mL) compared to those on DFO alone (4319 ng/mL) or the DFO+DFP combination (4180 ng/mL) (*P* = 0.0001 and *P* = 0.0021, respectively, Figure 3). Nevertheless, no statistically significant differences were observed in all paired comparisons of median SF concentrations between patients on other ICT regimens DFO vs. DFO+DFP (*P* = 0.585), DFO vs. DFP (*P* = 0.17), DFO+DFP vs. DFP (*P* = 0.296), and DFP vs. DFX (*P* = 0.207).

**Figure 4:**
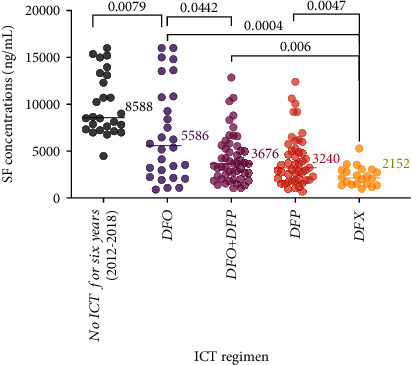
Comparison of SF concentrations (ng/mL) according to the administered ICT regimen in the (2019) cohort. The highest median SF concentration (8588 ng/mL) is observed in patients who received no ICT for six years (2012-2018), followed by patients on DFO (5586 ng/ml), DFO+DFP (3676 ng/ml), and DFP (3240 ng/ml), whereas the lowest median SF value (2152 ng/ml) was evident in patients receiving DFX.

Similarly, in the 2019 (during conflict) cohort, patients on DFX had a statistically significantly lower median SF concentration (2152 ng/mL) compared to those on DFO (5586 ng/mL), DFO+DFP (3676 ng/mL), or DFP (3240 ng/mL) (*P* = 0.0004, *P* = 0.0006, and *P* = 0.0047, respectively). On the contrary, patients on DFO had significantly higher SF concentrations than those on DFP or DFO+DFP. (*P* = 0.0251 and *P* = 0.0442, respectively). No statistically significant difference was observed between SF concentrations in patients receiving DFP versus those on DFO+DFP (*P* = .0215). Furthermore, all patients (*n* = 26, 100%) who did not receive ICT for six years (2012-2018) experienced severe iron overload evidenced by a median SF concentration of 8588 ng/mL and a range from 4481 and up to 16000 ng/mL, as illustrated in Figure 4.

### 3.4. SF Concentrations and Prevalence of Iron Overload in the Followed-up Cohort

**Figure 5:**
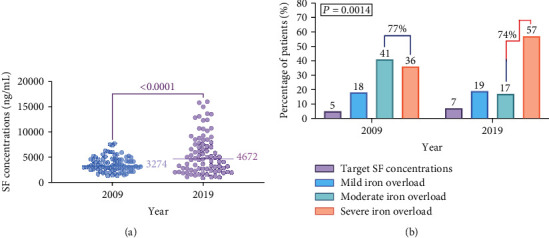
(a) A comparison of SF concentrations in the 2009 (preconflict) to 2019 (during conflict) followed-up patients (*n* = 84). (b) Prevalence of mild, moderate, and severe iron overload versus target SF concentrations in the followed-up cohort pre- (2009) and during (2019) the ongoing conflict.

Out of the 2009 (preconflict) cohort (*n* = 205), only 84 patients continued attending HNTC and were assessable in 2019. The median SF concentrations of the patients who were followed up significantly increased from 3274 ng/ml in 2009 to 4672 ng/ml in 2019 (*P* < 0.0001) (Figure 5(a)).

However, approximately similar low percentages (5% and 7%) of the followed-up patients reached the target SF concentrations in 2009 and 2019, respectively. It is worth noting that of the patients who were followed up from 2009 to 2019, a higher percentage (57%) experienced severe iron overload compared with 36% in 2019 and 2009, respectively (*P* = 0.0014, Figure 5(b)). Moreover, 25% of the followed-up patients demonstrated improvement in iron overload severity, while 39% and 36% experienced deterioration or no improvement, respectively.

## 4. Discussion

The Syrian conflict has been deemed by the United Nations as the most severe man-made catastrophe since World War II. Since its beginning in 2011, Syria's war has been the most complex multi-sided conflict since World War II, with devastating impacts on the lives of hundreds of thousands of Syrians [21, 22], including BTM patients. The limited availability and affordability of medicines, along with reduced accessibility to health facilities, have posed significant challenges for BTM patients in receiving indispensable health services, including ICT therapy. Despite these obstacles, the National Thalassemia Program in Syria has continued to provide free treatment to registered BTM patients, underscoring the importance of sustained support for vulnerable patient populations during times of crisis. As of May 2022, the Syrian Ministry of Health reported that 5830 BTM patients were registered at the National Thalassemia Program, with each patient receiving annually approximately 18 blood units as well as chelation therapy free of charge [20].

Our current study revealed a significantly elevated prevalence (98% and 89%) of iron overload among patients with BTM in Syria during the two study phases (i.e., 2009 and 2019). These findings emphasize the gap between the anticipated therapeutic outcomes of iron chelators and the real-world patterns and outcomes and the multitude of challenges faced by the National Thalassemia Program and BTM patients in Syria in the times of peace and conflict. Whereas poor adherence to chelation therapy due to a shortage of DFO pumps, especially in families with multiple BTM siblings, in addition to limited measures implemented to regular monitoring of SF concentration, have remained the main impediments prior to and during the Syrian conflict, violence and sanctions have aggravated these already difficult conditions. Nevertheless, our results also support pivotal roles played by HNTC, as well as other National Thalassemia Centers, in providing medical services to BTM patients, as evidenced by the high prevalence (100%) of severe iron overload in patients trapped in zones of armed conflict or caught in besieged territories who experienced unavailability and inaccessibility of iron chelators during the Syrian War.

Despite the challenges posed by the conflict, the medical staff at the HNTC has strived to maintain efficient provision of health services to BTM patients and improve BTM management outcomes by means of intensifying iron chelation therapy through combination regimens, improving adherence via replacing DFO with oral chelators (84% in 2019 vs. 16% in 2009), and implementing routine monitoring of SF concentrations. However, the increase in patients' age and, therefore, the cumulative number of transfused blood units received, together with the unavailability of some iron chelators during the conflict due to sanctions, contributed to the high prevalence of iron overload in the followed-up cohort.

The suboptimal outcomes of iron chelation therapy at HNTC also call for future studies on the cost-effectiveness of the four standard chelation regimens to support an evidence-based decision-making process regarding choosing an affordable and effective regimen in a low-income country exhausted by a twelve-year war. Our results prove that patients with BTM who were administered DFO monotherapy demonstrated highest SF concentrations, with levels exceeding 10000 ng/mL in nearly one-third of the patients in 2019. These results establish the inferiority of DFO in achieving target SF concentrations at both study time points (2009 and 2019). The primary challenge associated with DFO treatment is poor adherence due to the cumbersome, uncomfortable, inconvenient, and time-consuming nature of parenteral administration [15, 17]. The combination regimen appeared to provide enhanced chelation effectiveness compared to the standard subcutaneous DFO monotherapy but not to the oral DFP. Patients receiving DFX had the lowest SF concentrations in both cohorts. However, DFX was unavailable at the National Thalassemia Centers between 2018 and 2019, and only a small percentage of patients could afford it.

These findings diverge from a plethora of published evidence on the efficacy of DFO in reducing SF levels over the past thirty years. Prior research consistently demonstrated that regular administration of DFO is effective in lowering SF levels. However, our study is in agreement with findings by Di Maggio et al. that supported the superiority of the DFO and DFP combined therapy over DFO and DFP monotherapies in reducing SF concentrations [14]. On the contrary, a meta-analysis by Xia et al. [23] revealed that neither oral DFP monotherapy nor combined DFP and DFO therapy was significantly superior to DFO monotherapy in lowering SF levels in BTM patients [24]. Moreover, a meta-analysis comparing DFX with DFO and placebo by Dou et al. concluded that DFO monotherapy was better than low-dose DFX in lowering SF and liver iron concentration (LIC), whereas a high DFX dose (>30 mg/kg/day) was superior to DFO in lowering LIC [25].

The discrepancies between our study and others might be attributed to differences in the study designs and patient characteristics, as well as the impact of the ongoing conflict on the availability of iron chelators, which probably led to intermittent supply-dependent adherence.

Both DFP and DFX share the convenience and ease of the oral administration, which supports a higher compliance rate. However, DFP was inferior to DFX in both cohorts (2009 vs. 2019). Notably, the families of patients who received DFX hold a higher socioeconomic status and usually have better access to financial, educational, social, and health resources than those with a lower socioeconomic status. Therefore, they were more likely to show better compliance due to their awareness of the treatment regimen and complications resulting from BTM itself, as well as poor adherence to iron chelation.

The lack of psychological support for BTM patients at the HNTC center, as well as at other National Thalassemia Centers, may have also contributed to these nonfavorable outcomes. Our findings underscore the significance of personalized chelation therapy and the intensification of chelation regimens, particularly in patients with severe iron overload. Additionally, vigilant monitoring of SF concentration is recommended as an inexpensive tool to evaluate iron burden in low-income countries with limited resources. This study also advocates for the provision of psychological support to Syrian BTM patients, especially those who have experienced the turmoil of the armed conflict. This support is necessary to overcome not only the negative effects of thalassemia on self-esteem and self-confidence but also the consequences of the war on their mental health and well-being. Furthermore, our data demonstrate that the efforts of the Syrian MOH to provide free blood transfusions and iron chelators have achieved suboptimal therapeutic outcomes. Therefore, a multicomponent intervention, including psychological and educational interventions, is needed to enhance adherence.

This study has some limitations, including its being single-centered with a relatively small number of patients. Additionally, there was a loss to follow-up of a considerable number of patients from the 2009 cohort's due to the armed conflict, subsequent displacement, or death. Furthermore, the study has a retrospective design and therefore missing data from the patients' records was inevitable.

## 5. Conclusions

The present study sheds the light on the high prevalence of iron overload and low rates of favorable therapeutic outcomes in Syrian patients with BTM who received blood transfusions and ICTs at HNTC prior to and during the ongoing conflict. The suboptimal outcomes could be attributed to patients' inadequate compliance with DFO, the inaccessibility to some ICTs (such as DFX) due to sanctions, and the unavailability of ICTs in territories controlled by armed rebels. This study emphasizes the importance of raising awareness and education among healthcare providers and patients about the significance of adherence to chelation therapy and regular monitoring of SF concentrations. Additionally, it highlights the pivotal role of the local thalassemia care centers (such as HNTC) in providing medical services to BTM patients prior to and during the conflict and emphasizes the need for sustained support and funding for the National Thalassemia Program in Syria and other conflict-affected countries. In conclusion, this study illuminates the challenges and opportunities for enhancing the care and therapeutic outcomes of BTM patients in Syria.

## Figures and Tables

**Table 1:** Comparison of the patients' demographic and clinical characteristics between the 2009 (preconflict) and 2019 (during conflict) cohorts.

Characteristics	2009 (preconflict) cohort (*n* = 205)	2019 (during conflict) cohort (*n* = 172)	*P* value
Age (years)			
Median (Range)	14 (3-32)	10 (3-34)	**<0.0001** ^∗^
Sex, *n* (%)			
Males	102 (49.76%)	87 (50.58%)	0.8732
Females	103 (50.24%)	85 (49.42%)	
Number of blood units transfused per month	2-3 units	2-3 units	
Hb prior to blood transfusion Median (g/dL) (Range)	8.7 (7.2-9.5)	8.1 (6.7-9.9)	**<0.0001** ^∗^
Iron chelation regimen, *n* (%) Age (years), Median (Range)			
DFO	81 (39%)	28 (16.3%)	**0.0085** ^∗^
	13 (4-29)	16 (3-32)	
DFP	20 (10%)	47 (26.3%)	**0.0034** ^∗^
	9 (7-10)	12 (4-34)	
DFO+DFP	43 (21%)	50 (29.1%)	0.671
	15 (10-32)	15 (3-29)	
DFX	61 (30%)	21 (12.2)	0.1341
	7 (3-19)	6 (3-11)	
No ICT for six years (2012-2018)	0 (0%)	26 (15.1%)	—
		15 (9-29)	

^∗^ *p* value < 0.05 was considered statistically significant.

## Data Availability

The datasets used and/or analyzed during the current study are available from the corresponding author on reasonable request.

## Abbreviations

BTM: Beta thalassemia major
DFO: Deferoxamine
DFP: Deferiprone
DFX: Deferasirox
ICT: Iron chelation therapy
HbA: Absent hemoglobin a
HbF: Fetal hemoglobin
HNTC: Homs national thalassemia center
LIC: Liver iron concentration
MOH: Ministry of Health
SF: Serum ferritin.
